# Intravenous versus inhalational maintenance anaesthesia on postoperative recovery parameters after general anaesthesia: A randomized controlled study

**DOI:** 10.6026/973206300221361

**Published:** 2026-03-31

**Authors:** Leena Rohilla, Ritu Sharma, Rajeev Lochan Tiwari

**Affiliations:** 1 Department of Anaesthesia, Maharishi Chyawan Medical College, Koriawas Narnaul, Haryana, India; 2 Department of Anaesthesia, Fortis Escorts Hospital, Jaipur, Rajasthan, India

**Keywords:** Total intravenous anaesthesia (TIVA), inhalational anaesthesia, propofol, sevoflurane, postoperative recovery, QoR-15, postoperative nausea and vomiting (PONV)

## Abstract

Total intravenous anaesthesia (TIVA) using propofol-remifentanil versus sevoflurane maintenance affects postoperative recovery, PONV and patient outcomes in laparoscopic surgery, though evidence varies. Hence, this randomised trial compared TIVA (n=60) versus sevoflurane (n=60) in 120 ASA I-III adults undergoing elective laparoscopic cholecystectomy with standardised protocols. TIVA accelerated extubation (7.4 ±2.1 vs 9.6±2.5 min; p<0.001), Aldrete ≥9 recovery (23.1±4.8 vs 29.2±6.1 min; p<0.001) and shortened PACU stay (40 [35-45] vs 50 [42-60] min; p=0.002). TIVA reduced PONV incidence (20% vs 38.3%; p=0.03) with less rescue antiemetic use, while pain scores and opioid requirements stayed comparable between groups. Propofol-remifentanil TIVA optimises early recovery, lowers PONV and boosts 24-hour QoR-15 scores versus sevoflurane, positioning it as the superior choice for enhanced postoperative outcomes.

## Background:

Optimising the quality and speed of postoperative recovery is a key objective of modern anaesthetic practice, particularly in the era of day-case and fast-track surgery. Recovery encompasses not only physiological parameters, such as consciousness and cardiorespiratory stability, but also pain control, PONV, physical independence and emotional well-being [1, 2]. The Quality of Recovery-40 (QoR-40) and its abbreviated derivative, the QoR-15, are validated patient-reported outcome measures that capture these multidimensional aspects and are increasingly used as primary endpoints in perioperative research [3, 4-5]. Maintenance of general anaesthesia is commonly achieved either with volatile inhalational agents such as sevoflurane or desflurane or with propofol-based TIVA techniques. Pharmacokinetic and pharmacodynamic differences between these approaches, including context-sensitive half-times, tissue distribution and intrinsic antiemetic properties of propofol, could translate into different recovery profiles [6, 7- 8]. Early work suggested that propofol-based TIVA may reduce PONV and improve subjective recovery compared with volatile agents, though at the cost of slightly slower emergence in some settings [9, 10]. Several randomised trials have directly compared TIVA with volatile maintenance using global quality-of-recovery scales. Lee *et al.*reported improved QoR-40 scores with propofol-remifentanil TIVA versus desflurane after a range of non-cardiac surgeries [3]. Ahmed *et al.*observed higher QoR-40 scores with TIVA following endoscopic sinus and pituitary surgery, respectively [11]. In male patients undergoing lumbar surgery, Kim *et al.*found better QoR-40 with propofol-remifentanil compared with sevoflurane [8]. However, Park *et al.*demonstrated non-inferiority or only modest differences in QoR-15 or QoR-40 when comparing desflurane with propofol in ambulatory laparoscopic cholecystectomy and living kidney donation [12]. More recently, a meta-analysis by Shui *et al.* synthesized randomized controlled trials using QoR-40 as a primary endpoint and concluded that TIVA offers small but statistically significant improvements in early QoR-40 scores compared with volatile anaesthesia, although the certainty of evidence was low [4]. Systematic reviews of ambulatory surgery and bariatric surgery similarly suggest potential advantages of propofol-based TIVA for PONV and certain recovery parameters, but heterogeneity in patient populations, surgical procedures and outcome measures limits firm conclusions [13]. Therefore, it is of interest to compare propofol-based TIVA with sevoflurane-based inhalational maintenance anaesthesia in adults undergoing elective laparoscopic cholecystectomy.

## Materials and Methods:

## Study design and setting:

This was a single-centre, prospective, randomised, parallel-group controlled study conducted in the Department of Anaesthesiology at a tertiary care teaching hospital. Adult patients scheduled for elective laparoscopic cholecystectomy under general anaesthesia were enrolled over 12 months. The study protocol was approved by the Institutional Ethics Committee and written informed consent was obtained from all participants.

## Participants:s

Inclusion criteria were: age 18-65 years; ASA physical status I-III; scheduled for elective laparoscopic cholecystectomy; and ability to understand the QoR-15 questionnaire. Exclusion criteria were: anticipated difficult airway; severe hepatic, renal or cardiac dysfunction; history of malignant hyperthermia; chronic opioid or antiemetic use; psychiatric illness impairing questionnaire completion; pregnancy; morbid obesity (BMI >40 kg/m^2^); and known allergy to study drugs.

## Randomisation and blinding:

Participants were randomised in a 1:1 ratio to either the TIVA group (Group T) or the inhalational group (Group S) using a computer- generated random sequence with concealed, sequentially numbered opaque envelopes. Anaesthesiologists administering anaesthesia were aware of group allocation; however, PACU nurses, ward staff and the investigator collecting postoperative outcomes, including QoR-15, were blinded to group assignment.

## Anaesthetic technique:

All patients fasted according to institutional policy and received oral ranitidine 150 mg and alprazolam 0.25 mg on the night before surgery. In the operating room, standard monitoring (ECG, non-invasive blood pressure, pulse oximetry, capnography) and bispectral index (BIS) monitoring were instituted. Baseline haemodynamic variables were recorded. Anaesthesia was induced in both groups with intravenous fentanyl 2 µg/kg and propofol 2 mg/kg, followed by rocuronium 0.9 mg/kg to facilitate tracheal intubation. After intubation, lungs were ventilated with a 50% oxygen-air mixture targeting end-tidal CO_2_ 35-40 mmHg.

[1] Group T (TIVA): Anaesthesia was maintained with propofol infusion titrated to 75-150 µg/kg/min and remifentanil 0.05-0.20 µg/kg/min via target-controlled infusion pumps, adjusted to maintain BIS 40-60 and haemodynamic stability.

[2] Group S (sevoflurane): Anaesthesia was maintained with sevoflurane in oxygen-air (end-tidal 1.0-1.5 MAC) plus intermittent fentanyl boluses (0.5-1 µg/kg) as needed to maintain BIS 40-60 and stable haemodynamics.

Neuromuscular blockade was maintained with intermittent rocuronium guided by a peripheral nerve stimulator. All patients received intravenous paracetamol 15 mg/kg and intravenous diclofenac 1 mg/kg towards the end of surgery unless contraindicated. Ondansetron 4 mg IV was administered to all patients 20 min before the end of surgery as PONV prophylaxis. At the end of surgery, anaesthetic agents and infusions were discontinued. Neuromuscular blockade was reversed with neostigmine 0.05 mg/kg and glycopyrrolate 0.01 mg/kg. Extubation was performed when standard criteria were met.

## Postoperative management and outcome measures:

Patients were transferred to the PACU with standard monitoring. A modified Aldrete score was recorded every 5 min. Time intervals from discontinuation of anaesthetic to eye opening on verbal command, response to verbal command, extubation, achievement of Aldrete ≥9 and PACU discharge were documented. Pain was assessed using an 11-point numerical rating scale (NRS; 0=no pain, 10=worst). Intravenous morphine 2 mg boluses were administered for NRS ≥4 titrated to NRS ≤3. PONV was assessed using a 4-point ordinal scale (0=no nausea, 1=nausea, 2=vomiting, 3=severe refractory symptoms). Intravenous ondansetron 4 mg was given as a rescue antiemetic. Patients were followed up at 24 h postoperatively and the QoR-15 questionnaire was administered in their native language by a blinded investigator.

## Primary outcome:

Time (minutes) from cessation of anaesthetic agent/infusion to achieving a modified Aldrete score ≥9.

## Secondary outcomes:

[1] Time to eye opening and response to verbal command.

[2] Time to extubation.

[3] Duration of PACU stay (minutes).

[4] Incidence and severity of PONV within 24 h and rescue antiemetic requirements.

[5] NRS pain scores in PACU and at 24 h; total opioid consumption in morphine equivalents.

[6] QoR-15 score at 24 h postoperatively.

[7] Haemodynamic and intraoperative adverse events.

## Sample size calculation:

Based on previous studies demonstrating a 5-7 min difference in time to Aldrete ≥9 between TIVA and volatile anaesthesia with an SD of approximately 10 min, we considered a 6-min reduction clinically significant. To detect this difference with 80% power and α=0.05 (two-sided), 54 patients per group were required. Allowing for 10% attrition, 60 patients were enrolled in each group (total n=120).

## Statistical analysis:

Data were analysed using standard statistical software. Continuous variables were tested for normality (Shapiro-Wilk test). Normally distributed data are presented as mean ± SD and compared with an independent-samples t-test. Non-normally distributed data are presented as median (interquartile range) and compared with Mann-Whitney U test. Categorical variables are expressed as frequencies (percentages) and analysed using chi-square or Fisher's exact test as appropriate. A p-value <0.05 was considered statistically significant.

## Results:

Of 138 patients screened, 120 met the inclusion criteria and were randomised (Group T, n=60; Group S, n=60). No patient required conversion to open surgery or was lost to follow-up; thus, all were analysed per protocol. Baseline demographic variables, ASA grade, BMI and surgical duration were comparable between groups. The proportion of smokers and patients with diabetes or hypertension did not differ significantly (Table 1 - see PDF). The mean duration of anaesthesia and surgery was similar between groups. Mean BIS values remained within the target range in both groups. Total intraoperative opioid consumption, expressed in morphine equivalent units, was comparable. Mean arterial pressure and heart rate trends did not differ significantly; no patient experienced awareness or significant haemodynamic instability requiring study protocol deviation (Table 2 - see PDF). Group T exhibited significantly faster early recovery. Time to eye opening (6.3±1.9 versus 8.2±2.1 min; p<0.001), response to verbal command (6.9±2.0 versus 8.9±2.3 min; p< 0.001) and extubation (7.4±2.1 versus 9.6±2.5 min; p<0.001) were all shorter in Group T than in Group S. Most importantly, time to achieve an Aldrete score ≥9 was reduced in Group T (23.1±4.8 versus 29.2±6.1 min; p<0.001) and PACU length of stay was also shorter (median 40 [35-45] versus 50 [42-60] min; p=0.002) (Table 3, Figure 1 - see PDF). The proportion of patients meeting PACU discharge criteria within 45 min was higher in Group T (73.3% versus 46.7%; p=0.004) (Figure 2 - see PDF). NRS pain scores at PACU arrival and at 24 h were similar between groups (PACU: 4.1±1.4 versus 4.3±1.5, p=0.54; 24 h: 2.3±1.1 versus 2.5± 1.2, p=0.32), as were total opioid requirements (Table 4 - see PDF). However, PONV incidence within 24 h was significantly lower in Group T (20.0% versus 38.3%; p=0.03) and fewer patients required rescue antiemetic therapy (11.7% versus 28.3%; p=0.02). No patient experienced intractable vomiting or required unplanned admission for PONV. Mean QoR-15 score at 24 h was significantly higher in Group T (126±12) compared with Group S (118±14; p=0.001). Improvements were most notable in the domains of physical comfort and emotional state, consistent with earlier reports using QoR scales to compare anaesthetic techniques. No serious adverse events attributable to the anaesthetic technique occurred in either group. Table 1 (see PDF) demonstrates adequate comparability between the groups in demographic and baseline clinical variables. Age, sex distribution, BMI and ASA physical status were well matched, as were the prevalence of common comorbidities and mean surgical duration. The p-values indicate no statistically significant imbalances, supporting successful randomisation. Consequently, subsequent between-group differences in recovery parameters are unlikely to be attributable to confounding by baseline characteristics.

Table 2 (see PDF) shows that, apart from the expected differences in anaesthetic agents, intraoperative conditions were similar. Depth of anaesthesia was comparable, as indicated by BIS values and haemodynamic parameters remained stable in both groups. Opioid requirements did not differ significantly, suggesting that analgesic depth was equivalent. These findings support that observed differences in postoperative recovery are attributable primarily to the maintenance anaesthetic technique rather than intraoperative instability or unequal opioid exposure. Table 3 (see PDF) indicates that propofol-based TIVA was associated with consistently faster early emergence markers. Patients in Group T opened their eyes, obeyed commands and were extubated several minutes earlier than those receiving sevoflurane. This translated into earlier attainment of an Aldrete score ≥9 and shorter PACU length of stay, with a higher proportion of TIVA patients being discharge- ready within 45 minutes. These differences are statistically and likely clinically relevant in high-throughput surgical settings. Table 4 (see PDF) shows that analgesic outcomes were similar between groups, with no significant differences in pain scores or opioid consumption. In contrast, PONV incidence and rescue antiemetic use were significantly lower in the TIVA group, consistent with propofol's recognised antiemetic properties [9, 10]. Mean QoR-15 scores were also higher after TIVA, indicating better overall patient-perceived recovery at 24 h. Together, these findings suggest that TIVA improves global recovery quality without compromising postoperative analgesia. Figure 1 (see PDF) illustrates that patients receiving propofol-based TIVA achieved consistently faster early recovery than those maintained with sevoflurane. Times to eye opening, response to verbal command and tracheal extubation were all several minutes shorter in Group T. This advantage extends to functional recovery, as reflected by earlier attainment of an Aldrete score ≥9 and reduced PACU stay. Collectively, these findings suggest that TIVA facilitates more efficient post-anaesthesia throughput in fast-track surgical settings. The cumulative recovery curves in Figure 2 (see PDF) highlight that TIVA shifted the entire early recovery trajectory to the left, with more patients reaching functional recovery targets sooner. The separation between curves is most pronounced in the first 30-45 minutes, a critical window for PACU throughput and resource use. These data complement Table 3 (see PDF), underscoring the practical advantage of propofol-based TIVA in fast-track perioperative pathways.

## Discussion:

This randomised controlled trial demonstrates that propofol-based TIVA confers clinically relevant advantages over sevoflurane maintenance anaesthesia in adults undergoing elective laparoscopic cholecystectomy. TIVA was associated with faster early emergence, earlier achievement of Aldrete ≥9, shorter PACU stay, lower PONV incidence and higher QoR-15 scores at 24 h, while pain scores and opioid requirements remained comparable between groups. Our findings align with prior work demonstrating improved quality of recovery with TIVA in various surgical populations. Lee *et al.*reported significantly higher QoR-40 scores and improved patient satisfaction with propofol-remifentanil TIVA compared with desflurane in non-cardiac surgery [3]. Liu *et al.*showed that TIVA improved QoR-40 after endoscopic sinus and transsphenoidal pituitary surgery, respectively, with benefits particularly evident in physical comfort and emotional domains [7]. Similarly, Kim *et al.* found superior QoR-40 scores in male patients undergoing lumbar surgery with TIVA versus sevoflurane [8]. Our observation of reduced PONV with TIVA is consistent with multiple randomised trials and meta-analyses [10, 13]. Gupta *et al.*demonstrated small differences in early recovery times but lower antiemetic requirements in propofol-based techniques compared with volatile anaesthetics in ambulatory surgery [10]. Ahmed *et al.*[11] reported that TIVA reduced PONV and postoperative pain in bariatric surgery, although effects on discharge time were modest. In the broader literature, volatile anaesthetics are recognised risk factors for PONV, whereas propofol exerts intrinsic antiemetic effects, likely via modulation of serotonin and dopamine pathways and reduced emetogenic trigeminovascular activation [12]. Not all studies have shown a clear superiority of TIVA. Zaballos *et al.* found non-inferior QoR-15 scores for desflurane versus TIVA in ambulatory laparoscopic cholecystectomy [6]. A large systematic review by Shui *et al.* concluded that TIVA improves QoR-40 on the day of surgery but that differences attenuate by postoperative day 1, reflecting modest effect sizes [4]. Recent meta-analyses focusing on mortality and major morbidity suggest equivalence between techniques for hard outcomes [14].

Our study contributes to this evidence base by combining objective PACU metrics with a patient-reported QoR-15 endpoint within a standardised enhanced recovery protocol for a common laparoscopic procedure. The concordance between faster PACU recovery and improved QoR-15 suggests that TIVA's benefits extend beyond simple emergence speed to encompass subjective well-being and functional recovery. The magnitude of difference in QoR-15 (approximately 8 points) approaches or exceeds reported minimal clinically important differences for this scale, supporting its clinical relevance [15]. Several mechanisms may underlie the superiority of TIVA observed here. Propofol's rapid redistribution and context-sensitive half-time may facilitate smooth emergence, particularly when combined with short-acting opioids and goal-directed depth of anaesthesia monitoring. Its antiemetic action reduces PONV-related discomfort, dehydration and delays in oral intake. In contrast, volatile agents may contribute to residual dizziness, nausea and airway irritation, which could negatively affect early QoR scores. Additionally, emerging data suggest that propofol may attenuate neuroinflammatory responses compared with volatile agents, potentially influencing postoperative cognitive and emotional domains of QoR [16, 17]. Our trial has limitations. First, it was conducted at a single centre and restricted to relatively healthy adults undergoing laparoscopic cholecystectomy, which may limit generalizability to high-risk or different surgical populations. Second, anaesthesiologists could not be blinded to group allocation, introducing potential performance bias, although outcome assessors and patients rating QoR-15 were blinded. Third, while the sample size was adequate for detecting differences in early recovery times and QoR-15 scores, the study was not powered to evaluate rare adverse events or long-term outcomes. Finally, all patients received multimodal analgesia and standardised PONV prophylaxis; the observed differences may be attenuated or exaggerated in settings with different perioperative protocols. Future research should explore whether the benefits of TIVA on recovery persist in larger, multicentre cohorts, in high-risk elderly patients and within specific enhanced recovery pathways, including cardiac and neurosurgical populations. Comparative cost- effectiveness analyses incorporating drug acquisition costs, PACU staffing and unplanned admissions for PONV could clarify the economic implications of wider TIVA adoption [4, 13 and 15].

## Conclusion:

Propofol-based TIVA produces faster emergence, Aldrete ≥9 achievement, shorter PACU stay, lower PONV and higher QoR-15 scores versus sevoflurane maintenance in laparoscopic cholecystectomy, without compromising analgesia or haemodynamic stability. These superior recovery parameters establish anaesthetic maintenance technique as a clinically important, modifiable determinant of postoperative quality across emergence, antiemetic needs and patient-reported outcomes. TIVA should be prioritised for fast-track laparoscopic procedures, especially in PONV-prone patients or institutions optimising PACU throughput and patient-centered recovery metrics.
